# Identification of Powdery Mildew Responsive Genes in *Hevea brasiliensis* through mRNA Differential Display

**DOI:** 10.3390/ijms17020181

**Published:** 2016-01-29

**Authors:** Xiang Li, Zhenghong Bi, Rong Di, Peng Liang, Qiguang He, Wenbo Liu, Weiguo Miao, Fucong Zheng

**Affiliations:** 1 College of Environment and Plant Protection, Hainan Key Laboratory for Sustainable Utilization of Tropical Bioresource, Hainan University, No. 58, Renmin Avenue, Haikou 570228, China; lixiaoxiao1986@hotmail.com (X.L.); dirong@comcast.net (R.D.); jpdiary@163.com (P.L.); hqg11300817@163.com (Q.H.); saucher@hainu.edu.cn (W.L.); 2 College of Agriculture, Hainan University, Haikou 570228, China; bizhenghong9999999@163.com; 3 Department of Plant Biology and Pathology, Rutgers, the State University of New Jersey, 59 Dudley Road, New Brunswick, NJ 08901, USA

**Keywords:** rubber tree, *Oidium heveae*, differential gene expression, ESTs

## Abstract

Powdery mildew is an important disease of rubber trees caused by *Oidium heveae* B. A. Steinmann. As far as we know, none of the resistance genes related to powdery mildew have been isolated from the rubber tree. There is little information available at the molecular level regarding how a rubber tree develops defense mechanisms against this pathogen. We have studied rubber tree mRNA transcripts from the resistant RRIC52 cultivar by differential display analysis. Leaves inoculated with the spores of *O. heveae* were collected from 0 to 120 hpi in order to identify pathogen-regulated genes at different infection stages. We identified 78 rubber tree genes that were differentially expressed during the plant–pathogen interaction. BLAST analysis for these 78 ESTs classified them into seven functional groups: cell wall and membrane pathways, transcription factor and regulatory proteins, transporters, signal transduction, phytoalexin biosynthesis, other metabolism functions, and unknown functions. The gene expression for eight of these genes was validated by qRT-PCR in both RRIC52 and the partially susceptible Reyan 7-33-97 cultivars, revealing the similar or differential changes of gene expressions between these two cultivars. This study has improved our overall understanding of the molecular mechanisms of rubber tree resistance to powdery mildew.

## 1. Introduction

The rubber tree (*Hevea brasiliensis*) is the only economical natural source for natural rubber, which cannot be replaced by artificial synthetic polymers [1,2]. Powdery mildew caused by *Oidium heveae* B.A. Steinmann is a worldwide leaf disease affecting rubber tree growth [3]. *O. heveae* attacks immature leaves, causing defoliation and curling of leaves, growth retardation, and reduction in latex yield [4]. Presently chemical application is the main method to control this rubber tree disease, but it is time consuming and labor intensive. Using resistant cultivars is considered more effective and environmentally friendly than chemical application. Therefore, it is important to investigate genes related to powdery mildew defensive mechanisms in the rubber tree. Plants have developed a variety of defensive mechanisms against pathogen attacks [5]. In response to pathogen infection, various plant genes are regulated to resist the challenge from pathogens. Cloning host resistance genes will be helpful to understand the mechanism of plant resistance to pathogens at the molecular level and to develop resistant cultivars by the transgenic approach. Unfortunately, none of the resistance genes has been isolated from the rubber tree and there is little information available regarding the defense mechanisms of the rubber tree against *O. heveae* [3].

In this study, we conducted differential display analysis on a resistant rubber tree cultivar at different infection stages to evaluate the gene regulations at the transcriptome level and to identify differential gene expression changes from the interaction of resistant rubber tree with *O. heveae*. The Differential Display Reverse Transcriptase Polymerase Chain Reaction (DDRT-PCR) method was first reviewed in 1992 [6]. It later became one of the major tools to identify and clone genes that were differentially expressed at different states in a given tissue [7].

We then used the quantitative qRT-PCR method to validate and assess the expression changes of eight genes in two rubber tree cultivars: RRIC52, which is highly resistant; and Reyan 7-33-97, which is mildly susceptible to powdery mildew [8]. A resistant plant will rapidly activate gene expressions and produce defense responsive compounds to enhance or induce resistance and prevent pathogen colonization. We aimed to identify the responsive genes expressed during powdery mildew infection in the resistant cultivar RRIC52. The expression changes of these genes were also compared between the resistant RRIC52 and the susceptible Reyan 7-33-97 cultivars.

## 2. Results

### 2.1. Comparison of Powdery Mildew Resistance between RRIC52 and Reyan 7-33-97

**Figure 1:**
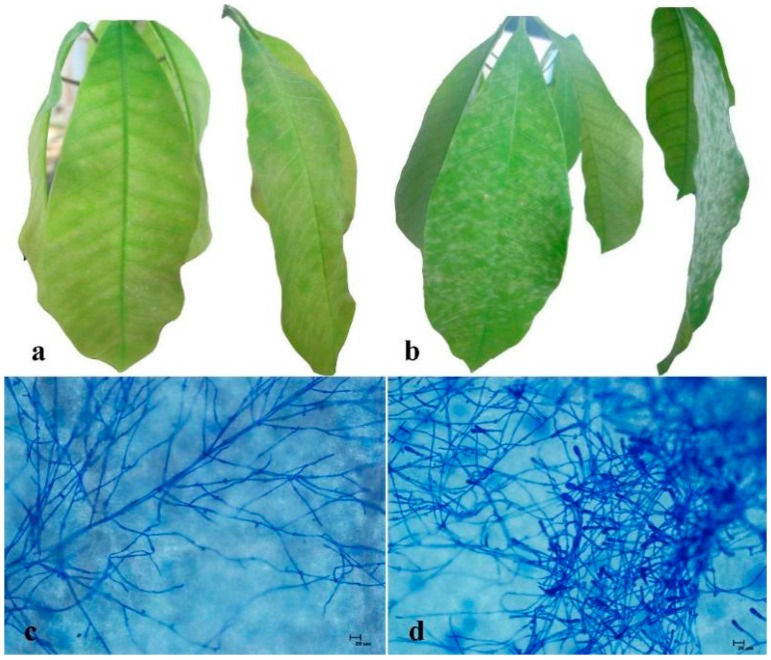
Disease resistance comparison between the highly resistant RRIC52 (**a**) and the mildly susceptible Reyan 7-33-97 (**b**) infected with powdery mildews at 120 h post inoculation; Microscopic observation of the powdery mildew hyphae on the leaves of RRIC52 (**c**) and Reyan 7-33-97 (**d**). Bar = 20 μm.

To compare the difference of disease resistance between RRIC52 and Reyan 7-33-97, both cultivars were infected with powdery mildew pathogen *O. heveae* (Figure 1). On Reyan 7-33-97 leaves, powdery mildew grew densely and the leaves were completely covered with the fungus after 120 h post inoculation (hpi). However, the white powdery colonies were not visible on the upper leaf surface of RRIC52, indicating its resistance to powdery mildew. Moreover, microscopic observation revealed fewer hyphae and conidiophores of powdery mildews produced on the leaf of RRIC52 than Reyan 7-33-97. These results indicate that RRIC52 was more resistant to *O. heveae* than Reyan 7-33-97.

### 2.2. Identification and Isolation of Differentially Expressed Transcripts

**Figure 2:**
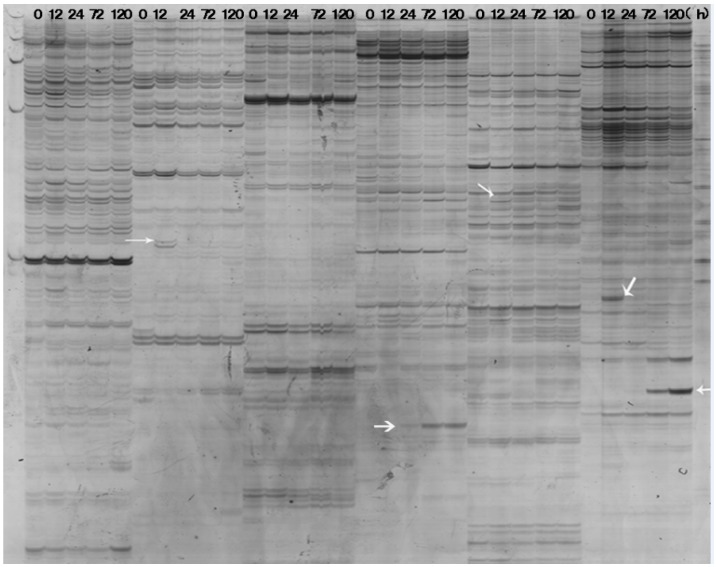
Polyacrylamide gel electrophoresis of partial differentially expressed DNAs (indicated by arrows) amplified by DDRT-PCR from the total RNA samples of RRIC52. Each lane indicates sample from 0, 12, 24, 72, or 120 hpi. Primer sets for each sample group from left to right are H-T11 A and H-AP27, H-T11 A and H-AP28, H-T11 A and H-AP29, H-T11 A and H-AP30, H-T11 A and H-AP30, and H-T11 A and H-AP30.

**Figure 3:**
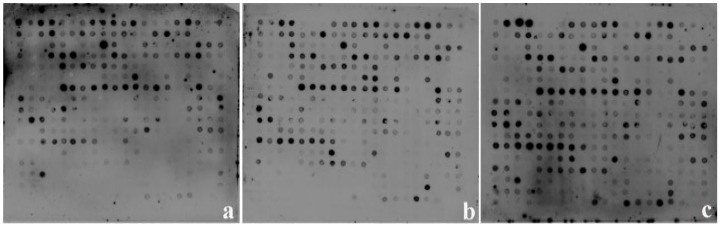

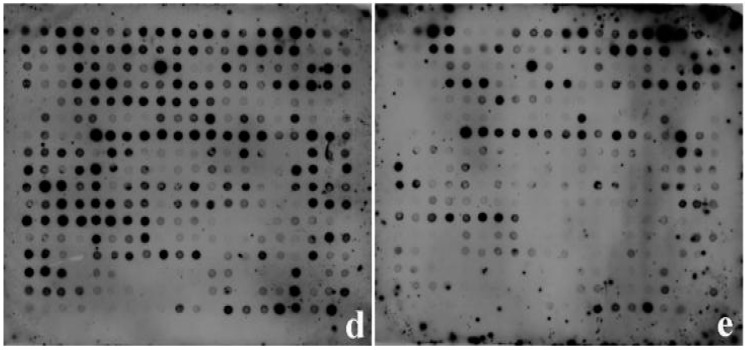
Reverse Northern dot blot analysis of differentially expressed genes. Cloned cDNAs were amplified, denatured, and blotted on five nylon membranes as described. Membranes were hybridized with DIG-labeled total cDNAs from control. (**a**) 0 hpi; (**b**) 12 hpi; (**c**) 24 hpi; (**d**) 72 hpi; (**e**) 120 hpi.

The DDRT-PCR was performed using random combinations of one base-anchored 3’-oligo (dT) primer and one arbitrary primer to amplify the single-stranded cDNAs produced from the total RNA samples of RRIC52 at 0 (control), 12, 24, 72, and 120 hpi, respectively. After separating on a 6% urea-polyacrylamide gel, the DNA bands up- or down regulated following pathogen infection were selected and excised from the gel (Figure 2). Approximately 300 differentially expressed DNA bands were selected and used as templates for the second round of PCR amplification. The second round of PCR produced single DNA bands (Supplementary Material, Figure S1) that were verified by reverse Northern dot blot hybridization with the probes synthesized from the RNA samples that were used in the differential display analysis (Figure 3).

### 2.3. Sequence Analysis Revealed the Significances of Differentially Expressed RRIC52 Transcripts

The EST bands from the DDRT-PCR reactions were selected and cloned into pMD19T (Takara, Kusatsu, Japan) cloning vector and transformed into competent DH5α cells. These ESTs were re-amplified and verified by reverse Northern dot blot hybridization. After sequencing, 78 unique ESTs were deposited in the EST database of GenBank [9] under the library accession number LIBEST-028598. The sequences of these 78 differentially expressed transcripts were compared with BLASTx and BLASTn searches against non-redundant protein sequences and nucleotide sequences in the available NCBI databases. The homology searches showed that these sequences were significantly similar to known genes of other plant species involved in stress response. These EST sequences were classified into seven functional groups: cell wall and membrane pathways, transcription factor and regulatory proteins, transporters, signal transduction, phytoalexin biosynthesis, other metabolism functions, and unclear ESTs. The results of this analysis of the detailed information are summarized in Table 1 and we have also tabulated 78 ESTs with their fragment lengths, the time point when they were excised, and the primer combination in Table S1 (Supplementary Material). Additionally, the mapping of 78 ESTs to the reference genome is supplied in Table S2 (Supplementary Material), and the redundancy for 78 ESTs by blastn (BLAST 2.2.29+, *E*-value = 1 × 10^−10^) is analyzed in Table S3 (Supplementary Material).

### 2.4. Characterization of the Differentially Expressed ESTs

In the library, eight ESTs are shown to be involved in cell wall and membrane pathways. These include transcripts for one pectin esterase, four chitinases, one β-1,3-glucanase, and two germin-like proteins.

Another eight ESTs are highly similar to the predicted transcription factor and regulatory genes, including three WRKY transcription factors, a rop guanine nucleotide exchange factor, a ferritin gene, an eukaryotic initiation factor, and a transcription factor.

There are also 10 ESTs involved in transport activities. These include one clathrin assembly protein and one clathrin light chain, two DNA-directed RNA polymerase II sequences, two glycosyl transferase genes, one flavonol 4’-sulfotransferase, and one lipid-transfer protein.

There are 16 ESTs identified as genes associated with signal transduction, including four serine-threonine protein kinase genes, six kinase protein genes, one signal transducer and transcription activator gene, and two membrane pathways genes.

Eleven ESTs are shown to be involved in phytoalexin biosynthesis. These include transcripts for cytochrome P450, chalcone synthase (CHS), senescence-related gene, UDP-glucosyltransferase, leucoanthocyanidin dioxygenase. 1-Aminocyclopropane-1-carboxylate oxidase, geranyl pyrophosphate synthase. acireductone dioxygenase, short chain alcohol dehydrogenase, and galactinol synthase family protein.

ESTs involved in other metabolic pathways include photosystem related protein, ribosomal protein, ribulose-1, 5-bisphosphate, and special function proteins, which are usually involved in the stress responses of plants.

**Table 1:** List of ESTs differentially expressed in the resistant RRIC52 following *O. heveae* infection and their similarity to known proteins determined by BLAST analysis.

ESTs	dbEST_Id	Accession No	Homology Found with	Homologous Species	Identity %	Description	*E*-Value
**ESTs in cell wall and membrane pathways**							
HBOH2	79640384	JZ822682	XM_002522813.1	*Ricinus communis*	85.47	pectinesterase-2 precursor, putative	2 × 10^−87^
HBOH3	79721589	JZ893198	U48687.1	*Castanea sativa*	78.07	endochitinase	3 × 10^−164^
HBOH5	79721590	JZ893199	AB367524.1	*Arabidopsis thaliana*	66	CERK1 mRNA for chitin elicitor receptor kinase 1	1.00 × 10^−100^
HBOH7	79640387	JZ822685	XM_002530816.1	*Ricinus communis*	84.47	glucan endo-1,3-beta-glucosidase precursor, putative	3 × 10^−149^
HBOH15	79721594	JZ8931203	XM_006375808.1	*Populus trichocarpa*	80.33	germin-like protein 1	6 × 10^−72^
HBOH66	79761733	JZ897003	XM_006376356.1	*Populus trichocarpa*	81.84	class IV chitinase family protein	2 × 10^−132^
HBOH67	79761734	JZ897004	XM_002532766.1	*Ricinus communis*	80.94	chitinase 1 precursor, putative	3 × 10^−118^
HBOH68	79761735	JZ897005	AY973617.1	*Manihot esculenta*	88.24	germin-like protein GLP partial CdS	2 × 10^−163^
**ESTs involved in transcription factor and regulatory proteins**							
HBOH4	79640385	JZ822683	XM_002526622.1	*Ricinus communis*	86.35	ferritin, plant, putative	7 × 10^−171^
HBOH13	79721592	JZ8931201	XM_002303816.2	*Populus trichocarpa*	78.24	WRKY transcription factor 40 family protein	3 × 10^−85^
HBOH14	79721593	JZ8931202	XM_012596852.1	*Gossypium raimondii*	58	WRKY transcription factor 18-like	7 × 10^−15^
HBOH31	79761701	JZ896971	XM_002534015.1	*Ricinus communis*	87.56	eukaryotic initiation factor iso-4F subunit p82-34, putative	3 × 10^−62^
HBOH33	79761703	JZ896973	XM_002517578.1	*Ricinus communis*	78.79	DNA binding protein, putative	2 × 10^−140^
HBOH36	79761705	JZ896975	XM_002527643.1	*Ricinus communis*	84.82	transcription factor btf3, putative	0
HBOH55	79761724	JZ896994	XM_002524212.1	*Ricinus communis*	82.11	rop guanine nucleotide exchange factor, putative	0
HBOH60	79761727	JZ896997	XM_006368449.1	*Populus trichocarpa*	85.21	WRKY transcription factor 40 family protein	4 × 10^−140^
**ESTs involved in transporter**							
HBOH8	79640388	JZ822686	XM_002521455.1	*Ricinus communis*	85.71	dimethyladenosine transferase, putative	1 × 10^−53^
HBOH28	79761698	JZ896968	XM_002527741.1	*Ricinus communis*	88.11	clathrin assembly protein, putative	2 × 10^−144^
HBOH38	79761707	JZ896977	XM_002529119.1	*Ricinus communis*	82.69	DNA-directed RNA polymerase II, putative	6 × 10^−154^
HBOH39	79761708	JZ896977	XM_002529119.1	*Ricinus communis*	83.15	DNA-directed RNA polymerase II, putative	3 × 10^−158^
HBOH44	79761713	JZ896983	XM_002269429.2	*Vitis vinifera*	66	clathrin light chain 2	5 × 10^−73^
HBOH46	79761715	JZ896985	XM_002531006.1	*Ricinus communis*	76.31	transferase, transferring glycosyl groups, putative	4 × 10^−90^
HBOH52	79761721	JZ896991	HM461981.1	*Vernicia fordii*	84.48	glycerol-3-phosphate acyltransferase 9 (GPAT9) gene	0.00003
HBOH54	79761723	JZ896993	HQ285842.1	*Hevea brasiliensis*	98.95	chloroplast, complete genome	0
HBOH75	79761741	JZ897011	XM_002524326.1	*Ricinus communis*	72.5	flavonol 4'-sulfotransferase, putative	4 × 10^−51^
HBOH81	79761747	JZ897017	XM_007017019.1	*Theobroma cacao*	72.69	non-specific lipid-transfer protein, putative	2 × 10^−26^
**ESTs involved in signal transduction**							
HBOH1	79640383	JZ822681	XM_002516001.1	*Ricinus communis*	68.49	serine-threonine protein kinase, plant-type, putative	4 × 10^−63^
HBOH6	79640386	JZ822684	XM_002324361.2	*Populus trichocarpa*	87.66	H^+^-ATPase family protein mRNA, complete cds	2 × 10^−130^
HBOH9	79721591	JZ893120	XM_003605902.1	*Medicago truncatula*	40	hypothetical protein mRNA, complete cds	0.0001
HBOH10	79640389	JZ822687	XM_007019829.1	*Theobroma cacao*	86.79	cysteine-rich RLK (RECEPTOR-like protein kinase) 8	0.00002
HBOH22	79761693	JZ896963	XM_002514747.1	*Ricinus communis*	87.7	4-nitrophenylphosphatase, putative	0
HBOH23	79761694	JZ896964	XM_007019349.1	*Theobroma cacao*	66	cysteine-rich RLK (RECEPTOR-like protein kinase) 8	5 × 10^−43^
HBOH26	79761697	JZ896967	XM_002509773.1	*Ricinus communis*	83.05	ATP binding protein, putative	3 × 10^−170^
HBOH41	79761710	JZ896980	XM_002308527.2	*Populus trichocarpa*	71.47	kinase family protein	9 × 10^−35^
HBOH42	79761711	JZ896981	AM478326.2	*Vitis vinifera*	84.55	contig VV78X154662.12, whole genome shotgun sequence	5 × 10^−24^
HBOH45	79761714	JZ896984	XM_007026459.1	*Theobroma cacao*	95.35	signal transducer and transcription activator isoform 1	1 × 10^−28^
HBOH53	79761722	JZ896992	XM_002524179.1	*Ricinus communis*	75.7	kinase, putative	3 × 10^−10^
HBOH56	79761725	JZ896995	XM_002524179.1	*Ricinus communis*	75.26	kinase, putative	6 × 10^−47^
HBOH73	79761739	JZ897009	XM_002531397.1	*Ricinus communis*	83.52	serine-threonine protein kinase, plant-type, putative	1 × 10^−74^
HBOH74	79761740	JZ897010	XM_002523439.1	*Ricinus communis*	82.36	serine-threonine protein kinase, plant-type, putative	5 × 10^−139^
HBOH78	79761744	JZ897014	XM_002530595.1	*Ricinus communis*	69.87	B-Raf proto-oncogene serine/threonine-protein kinase, putative	9 × 10^−27^
HBOH80	79761746	JZ897016	XM_002513521.1	*Ricinus communis*	88.85	kinase, putative	3 × 10^−107^
**ESTs involved in phytoalexin biosynthesis**							
HBOH48	79761717	JZ896987	DQ371802.1	*Populus alba*	83.36	chalcone synthase (CHS) gene, CHS-M2 allele	5 × 10^−159^
HBOH61	79761728	JZ896998	XM_002300417.2	*Populus trichocarpa*	78.07	senescence-related gene 1 family protein	5 × 10^−180^
HBOH62	79761729	JZ896999	XM_002517028.1	*Ricinus communis*	85.4	acireductone dioxygenase, putative	6 × 10^−35^
HBOH63	79761730	JZ897000	XM_002519376.1	*Ricinus communis*	84.65	UDP-glucosyltransferase, putative	0
HBOH70	79761737	JZ897007	XM_002533589.1	*Ricinus communis*	83.4	leucoanthocyanidin dioxygenase, putative	0
HBOH72	79761738	JZ897008	XM_002524028.1	*Ricinus communis*	76.42	short chain alcohol dehydrogenase, putative	4 × 10^−98^
HBOH76	79761742	JZ897012	AY207387.1	*Hevea brasiliensis*	98.12	1-aminocyclopropane-1-carboxylate oxidase	5 × 10^−98^
HBOH77	79761743	JZ897013	XM_012216610.1	*Jatropha curcas*	99	cytochrome P450 82C4-like (LOC105633910), mRNA	1 × 10^−61^
HBOH79	79761745	JZ897015	XM_002311738.2	*Populus trichocarpa*	84.97	galactinol synthase family protein	0
HBOH83	79761749	JZ897019	XM_002531145.1	*Ricinus communis*	81.68	geranyl geranyl pyrophosphate synthase, putative	2 × 10^−146^
HBOH85	79761751	JZ897021	XM_002531048.1	*Ricinus communis*	100	cytochrome P450, putative, mRNA	1 × 10^−81^
**ESTs involved in other metabolism**							
HBOH11	79640390	JZ822688	XM_002512371.1	*Ricinus communis*	77.2	histone h2a, putative	3 × 10^−59^
HBOH12	79640391	JZ822689	XM_002297827.2	*Populus trichocarpa*	84.94	Humj1 family protein	2 × 10^−124^
HBOH18	79761690	JZ896960	HM363448.1	*Hevea brasiliensis*	94.58	60S ribosomal protein L27B (RPL27B)	3 × 10^−143^
HBOH19	79761691	JZ896961	XM_007008726.1	*Theobroma cacao*	85.13	ubiquitin-specific protease 24 isoform 4	1 × 10^−53^
HBOH29	79761699	JZ896969	XM_002519479.1	*Ricinus communis*	71.98	ribonucleoprotein, chloroplast, putative	8 × 10^−28^
HBOH30	79761700	JZ896970	KC533451.1	*Jatropha curcas*	70.7	SCAR marker SCAR27-740-NT genomic sequence	3 × 10^−16^
HBOH32	79761702	JZ896972	XM_003540551.2	*Glycine max*	76.5	RNA-binding protein 24-like	3 × 10^−59^
HBOH40	79761709	JZ896979	XM_002520681.1	*Ricinus communis*	85.64	photosystem I reaction center subunit II, chloroplast precursor, putative	0
HBOH47	79761716	JZ896986	AY247789.1	*Hevea brasiliensis*	72.73	HEV2.1 gene	1 × 10^−31^
HBOH49	79761718	JZ896988	XM_002512371.1	*Ricinus communis*	80.46	histone h2a, putative	7 × 10^−68^
HBOH57	79761726	JZ896996	M60274.1HEVRBSS	*Hevea brasiliensis*	100	ribulose-1,5-bisphosphate carboxylase small subunit (RbsS)	7 × 10^−89^
HBOH64	79761731	JZ897001	HQ285842.1	*Hevea brasiliensis*	100	chloroplast, complete genome	3 × 10^−171^
HBOH84	79761750	JZ897020	HG975448.1	*Solanum pennellii*	82.22	chromosome ch09, complete genome	4 × 10^−28^
**Unclear ESTs**							
HBOH17	79761689	JZ896959	XM_002321005.2	*Populus trichocarpa*	73.09	hypothetical protein	2 × 10^−24^
HBOH21	79761692	JZ896962	XM_002529035.1	*Ricinus communis*	84.38	conserved hypothetical protein	3 × 10^−107^
HBOH24	79761695	JZ896965	XM_002514837.1	*Ricinus communis*	73.53	conserved hypothetical protein	6 × 10^−90^
HBOH25	79761696	JZ896966	CT028466.1	*Poplar*	78.79	cDNA sequences	7 × 10^−24^
HBOH35	79761704	JZ896974	FN552731.1	*Hevea brasiliensis*	90	microsatellite DNA, clone mHbCIRA2697	2 × 10^−51^
HBOH37	79761706	JZ896976	XM_002519498.1	*Ricinus communis*	82.3	conserved hypothetical protein	0
HBOH43	79761712	JZ896982	HQ285842.1	*Hevea brasiliensis*	99.72	chloroplast, complete genome	0
HBOH50	79761719	JZ896989	XM_002517090.1	*Ricinus communis*	86.52	protein FRIGIDA, putative	3 × 10^−168^
HBOH51	79761720	JZ896990	EF146205.1	*Populus trichocarpa*	69.32	clone WS0116_D21 unknown mRNA	2 × 10^−12^
HBOH65	79761732	JZ897002	AY439300.1	*Hevea brasiliensis*	83.91	clone hmct18 microsatellite sequence	2 × 10^−15^
HBOH69	79761736	JZ897006	XM_002519826.1	*Ricinus communis*	87.7	conserved hypothetical protein	2 × 10^−106^
HBOH82	79761748	JZ897018	AC213413.1	*Populus trichocarpa*	79.66	clone POP014-E15, complete sequence	7 × 10^−19^

### 2.5. Analysis of Selected EST Gene Expression Profiles in RRIC52 and Reyan 7-33-97 after Powdery Mildew Infection Using qRT-PCR

**Figure 4:**
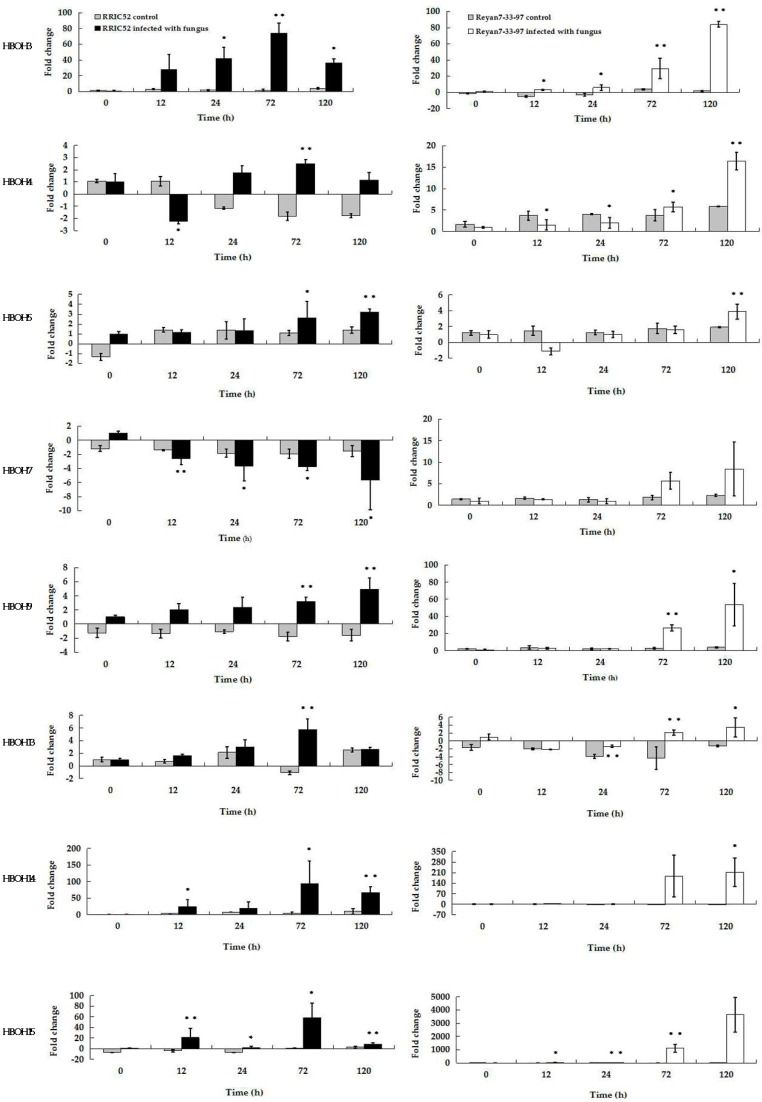
qRT-PCR analysis of eight EST expression levels in RRIC52 and Reyan 7-33-97 leaves during powdery mildew infection. Leaf samples were collected at 0, 12, 24, 72 and 120 hpi. Each gene expression level during the course of infection was compared to that at 0 hpi by the Pfaffl method [10], using *RH8* and *YLS8* in rubber tree as the reference genes; leaf samples from uninoculated plants of the same age were used as controls for each time point. Three biological replicates per cultivar were averaged and statistically analyzed using Student’s *t*-test (* indicates *p* < 0.05. **, *p* < 0.01) between control and powdery mildew-inoculated leaves. Bars indicate ± S.E. from the results of three biological replicates.

To understand the interaction between rubber tree and powdery mildew, we selected the first 15 ESTs (HBOH1-15) and analyzed their gene expression profiles in both the resistant RRIC52 and the mildly susceptible Reyan 7-33-97 using qRT-PCR. The leaves were harvested at 0, 12, 24, 72 and 120 h after powdery mildew infection, with three independent biological replicates at each time point. The gene expression in the inoculated plants was compared to that in the uninoculated plants of same developmental stages. Our results indicated that the expression of HBOH 1, 2, 6, 8, 10, 11, 12 ESTs less than doubled (data not shown). On the other hand, the results (Figure 4) showed that the expression levels of eight ESTs (HBOH 3, 4, 5, 7, 9, 13, 14, 15) were highly upregulated during the course of powdery mildew infection compared to the uninoculated control plants. However, the expression patterns of these eight ESTs greatly varied between RRIC52 and Reyan 7-33-97.

HBOH3, HBOH14, and HBOH15 were upregulated in both RRIC52 and Reyan 7-33-97, but the expression levels peaked at different times post infection. Our results showed that the upregulation of HBOH3, HBOH14, and HBOH15 expressions peaked at 72 hpi and then dropped in RRIC52 (Figure 4). However, the expression of these genes peaked at the later 120 hpi in Reyan 7-33-97, indicating a slower response of these genes in the susceptible cultivar. HBOH3 was predicted to be class 1 chitinase, catalyzing the hydrolysis of chitin polymers on a fungal cell wall. The expression of HBOH3 increased to 74.0-fold at 72 hpi in RRIC52, and to 84.7-fold at 120 hpi in Reyan 7-33-97. HBOH14 was predicted to be WRKY transcription factor, involved in the regulation of the immune effector process. The expression level of HBOH14 reached the highest of 94.6-fold at 72 hpi in RRIC52, and 213.5-fold at 120 hpi in Reyan 7-33-97. HBOH15 was predicted to be a germin-like protein, involved in plant cell wall reinforcement and papillae formation. Its expression level reached a peak of 58.3-fold at 72 hpi in the resistant RRIC52. In the mildly susceptible Reyan 7-33-97, the HBOH15 expression level peaked at 3657.3-fold. Another upregulated gene was HBOH9 (a hypothetical protein). In both rubber tree cultivars, it showed the same expression pattern. At 120 hpi, the expression levels of HBOH9 peaked at 5.0-fold in RRIC52 and 53.3-fold in Reyan 7-33-97.

HBOH5 and HBOH13 were both upregulated in RRIC52. The expression levels of HBOH5 (predicted to be chitin elicitor receptor kinase) reached a maximum at 120 hpi to 3.2-fold and HBOH13 (a WRKY transcription factor) reached a maximum at 72 hpi to 5.8-fold in RRIC52. In Reyan7-33-97, the expression level of HBOH5 decreased by 1.13-fold at 12 hpi, but then increased and reached a maximum at 120 hpi of 3.9-fold. The expression level of HBOH13 was first downregulated and then upregulated to 3.5-fold at 120 hpi.

HBOH4 was identified as a ferritin gene, regulating cellular iron under oxidative stress. Its expression level decreased 2.2-fold at 12 hpi, but went up to 2.5-fold at 72 hpi in RRIC52. In Reyan7-33-97, it increased 16.4-fold at 120 hpi. HBOH7 was upregulated in Reyan 7-33-97 but not in RRIC52. HBOH7 was predicted to be putative glucan endo-1,3-β-glucosidase precursor. Its expression was 5.7-fold downregulated at 120 hpi compared to 0 hpi in the resistant RRIC52. However, its expression level in the mildly susceptible Reyan 7-33-97 continued to increase as infection progressed and it reached 84.7-fold at 120 hpi.

**Table 2:** Sequence homology of differential display-PCR clones to sequences in the GenBank database.

Clone_ID	Primer Pair Used in DDRT-PCR	Size (bp)	Similar Genes Found in the Database	Functional Relevance	Plant Defense Mechanism Strategies
HBOH3	T11A/B0308	759	endochitinase (*Castanea sativa*)	Chitinases are enzymes that catalyze the hydrolysis of the beta-1,4-*N*-acetyl-d-glucosamine linkages in chitin polymers	catalyzes the hydrolysis of chitin polymers on fungal cell wall
HBOH4	T11G/B0314	685	ferritin, plant, putative, mRNA (*Ricinus communis*)	iron ion binding	regulation of cellular iron under oxidative stress
HBOH5	T11A/B0314	895	CERK1 mRNA for chitin elicitor receptor kinase 1 (*Arabidopsis thaliana*)	involved in receptor and signaling pathways, regulating innate immune responses and inflammation; involved in plant resistance to pathogen infection and herbivore attack	catalyzes the hydrolysis of chitin polymers on fungal cell wall
HBOH7	T11C/B0304	494	Glucan endo-1,3-β-glucosidase precursor, putative (*Ricinus communis*)	involved in carbohydrate binding	hydrolyses β-1,3 gucans on fungal cell wall
HBOH9	T11G/B0313	362	hypothetical protein mRNA (*Medicago truncatula*)	a single-pass membrane protein, sphingolipids play important roles in regulating cellular responses, involved in signaling pathways	signal transduction
HBOH13	T11C/B0322	525	WRKY transcription factor 40 family protein (*Populus trichocarpa*)	involved in the regulation of various physiological programs that are unique to plants, including pathogen defense, senescence and trichome development	regulation of immune effector process
HBOH14	T11G/B0308	251	WRKY transcription factor 18-like (*Gossypium raimondii*)	involved in the regulation of various physiological programs that are unique to plants, including pathogen defense, senescence and trichome development	regulation of immune effector process
HBOH15	T11A/AP34	305	germin-like protein 1 (*Populus trichocarpa*)	involved in plant cell wall reinforcement and papillae formation	protected epidermal cells from attack by fungus

## 3. Discussion

Plants defend themselves against pathogen infection through a variety of mechanisms at different stages during the pathogen infection. Of the powdery mildew resistance mechanisms that have been extensively studied, the barley *mlo* confers effective broad-spectrum resistance against all known isolates of *Blumeria graminis* f.sp. *hordei* (Bgh) [11]. The Run1 (Resistance to *Uncinula necator* 1) [12,13] and Ren1 (Resistance to *Erysiphe necator* 1) [14,15] are two grape loci associated with powdery mildew resistance. In Arabidopsis, the R gene-mediated defense came from the isolation and functional characterization of RPW8 conferring broad spectrum resistance to powdery mildew pathogens [16]. However, up to now, there have been very few studies on the rubber tree defensive mechanisms to *O. heveae* powdery mildew infection.

In this study, we used the DDRT–PCR method to identify genes that were differentially expressed in rubber tree resistant cultivar RRIC52 at five crucial stages during the powdery mildew infection. These five stages were identified as: (i) 0 h without infection as control; (ii) the 12 hpi stage with the primary haustoria formation reaching its peak; (iii) the 24 hpi stage with the pathogen forming hyphae from conidiospores; (iv) the 72 hpi stage with hyphae expanding on the leaf surface; and (v) the 120 hpi stage with lesion formation and fungal sporulation. Our results showed that at least 78 expressed sequence tags (ESTs) exhibited differentially regulated expressions in the interaction between rubber tree and *O. heveae* powdery mildew. The differences in the expression of several genes were analyzed by qRT-PCR and compared between the resistant RRIC52 and the susceptible Reyan 7-33-97 cultivars. This study improved our understanding of the gene network that regulates the interaction between *O. heveae* powdery mildew and rubber trees.

### 3.1. Cell Wall Pathway and PR Protein

Plant cell walls provide a physical barrier to pathogens. Plant cells have many mechanisms responding to cell wall damage. Germins and germin-like proteins (GLPs) are plant cell wall glycoproteins, classified into an exclusive cupin subfamily [17]. GLPs have been implicated in plant cell wall reinforcement and papillae formation during pathogen attack [18,19,20,21]. HBOH15 identified in our study matched to a germin-like protein in *P. trichocarpa*. Our data showed that its expression was upregulated >3000-fold in the mildly susceptible Reyan 7-33-97. It has been previously reported that several GLP subfamilies were strongly induced during *Blumeria graminis* f. sp. *hordei* infection, and the transient overexpression of GLPs protected barley epidermal cells from attack by the appropriate powdery mildew fungus [22]. *VvGLP3*, a GLP gene in grapevines, was induced specifically by *E. necator* infection, and involved in the defense response against this powdery mildew [23].

In addition, many fungi are thought to produce cell-wall-degrading enzymes in order to breach the host cell wall. Plants combat fungal infections by synthesis of a number of pathogenesis-related (PR) proteins [24]. One such example is β-1,3-glucanase. HBOH7 was found to be similar with the genes coding for β-1,3-glucanase in *Populus trichocarpa*. β-1,3-glucanase hydrolyzes β-glucans, which is a major component of fungal cell walls [25]. Another example is chitinase, an enzyme that catalyzes the hydrolysis of chitin polymers in the cell walls of invading fungal hyphae [26]. In our study the sequences of two DD-obtained transcripts, HBOH3 and HBOH5, were found to be similar to the genes coding for endochitinase of *Castanea sativa* and chitin elicitor receptor kinase 1 of *Arabidopsis thaliana*, respectively. Previous studies reported that both chitinase and β-1,3-glucanase activity were increased in grapevines against the powdery mildew fungus *U. necator* [27]. Our results showed these two ESTs were induced in both RRIC52 and Reyan 7-33-97 cultivars.

### 3.2. Transcription Factor

Chitin elicitation appears to play a significant role in plant defense to fungal pathogens. Chitin-responsive transcription factor genes likely represent key elements in the ability of chitin to modify gene expression as part of the plant defense reaction. It has been reported that there were 14 WRKY genes significantly upregulated after chitin treatment in Arabidopsis [28]. WRKY factors are key regulators in modulating the expression of genes involved in plant defense response pathway [29]. In our study, HBOH13 and HBOH14 were induced upon infection with the powdery mildew during early stages of infection. They were identified as WRKY18 and WRKY40, respectively. WRKY18 and WRKY40 are thought to negatively regulate resistance towards the biotrophic powdery mildew fungus *Golovinomyces orontii* [30,31]. In barley, the transcriptional knockdown of *HvWRKY1 HvWRKY2* that share the highest homology to Arabidopsis *WRKY18* and *WRKY40* results in an increased resistance against *Blumeria graminis* f. sp. *hordei* (Bgh) [32].

### 3.3. Transporter

Endocytosis in plants has an essential role not only for basic cellular functions but also for pathogen defense. The major endocytic mechanism in plants is clathrin-mediated endocytosis [33]. This study identified two ESTs, HBOH28 (similar to clathrin assembly protein) and HBOH44 (similar to clathrin light chain), that may participate in this pathway. HBOH55 was identified as being among the guanine nucleotide exchange factors (GEFs) that are known to be required for endocytosis and play a role in clathrin-dependent endocytosis in plants [34].

Plant lipid transfer proteins (LTPs) targeted to epidermal cell walls [35] are reported to be induced in response to pathogen infection and are included as PR-proteins [36]. HBOH81 was identified as a lipid-transfer protein gene. When an LTP (Ace-AMP1) isolated from *Allium cepa* was introduced into wheat plants, it enhanced transgenic wheat plants’ resistance to powdery mildew *Blumeria graminis* f. sp. *tritici* [37]. When the same gene was transplanted into roses, it also conferred resistance to powdery mildew *Sphaerotheca pannosa* [38].

### 3.4. Signal Transduction

ESTs involved in signal transduction pathways included some plant protein kinases. Certain protein kinases were likely to be crucially associated with plant immune responses. Our study showed that a few protein kinases were induced by powdery mildew in rubber tree: HBOH41, HBOH53, HBOH56, and HBOH80, which were identified as protein kinases. HBOH1, HBOH42, HBOH73, HBOH74, and HBOH78 were similar to serine-threonine protein kinase, and HBOH45 was identified as a signal transducer and transcription activator.

Previous study has shown that a putative serine and threonine protein kinase gene from *H. villosa* (Stpk-V) was significantly upregulated by Bgt (*Blumeria graminis* f. sp. *tritici*). The transformation of Stpk-V into a susceptible wheat variety conferred high and broad-spectrum resistance to powdery mildew [39]. The Pto kinase contained similar domains as serine/threonine (S/T) kinases, conferred resistance to bacterial-speck disease in tomato plants [40]. Protein kinases are also involved in the development of HR, SAR, and the SA-mediated induction of PR-1 gene expression in tobacco [41].

### 3.5. Phytoalexin Biosynthesis

Plants respond to pathogen attacks by the synthesis of not only pathogenesis-related (PR) proteins, such as glucanases and chitinases, but also antimicrobial compounds, such as phytoalexins [42]. Phytoalexin biosynthesis genes were identified in our study, indicating the importance of phytoalexins in the defense response of rubber tree to powdery mildew infection. The phenylpropanoid pathway is central in the plant defense response [43]. HBOH48 encodes a chalcone synthase (CHS) gene. CHS is a key enzyme in the phenylpropanoid pathway; its enhanced expression indicates the activation of the resistant response [44]. CHS can induce plants to produce more flavonoids and other related metabolites against stress [45]. Flavonoid-type phytoalexins are important in protecting plants from various phytopathogens and stress [46,47], and preformed flavonoids in host–pathogen interactions may play a direct signaling role in defense [48]. In our study, four ESTs (HBOH48, HBOH61, HBOH63, and HBOH70) were found to be involved in the flavonoid biosynthetic pathway.

Two cytochrome P450s ESTs (HBOH77, HBOH85) were identified in our study. Cytochrome P450s, a family of hemoproteins, play a prominent role in plant defense as they are instrumental in the biosynthesis of terpenoid, lignins, alkaloids, and a variety of other secondary components that are common defense agents [49,50,51]. A novel cytochrome P450, CaCYP1, was isolated in pepper plants and shown to play a potential role in plant defense response pathways that involve ABA- and SA-mediated signaling mechanisms [52]. A *Phytophthora infestans*-induced cytochrome P450 gene was potentially associated with quantitative resistance to potato late blight disease [43]. In Arabidopsis, cytochrome P450 genes were shown to be upregulated after inoculation with *A. brassicicola* and *A. alternata* [53].

### 3.6. ESTs Involved in Other Metabolic Pathways 

HBOH4 was shown to share high homology with ferritins. Its expression reached the highest level at 72 hpi in the resistant RRIC52. It has been reported that ferritin enhanced tolerance to ROS (reactive oxygen species) and necrosis caused by viral and fungal infections in tobacco [54]. The *Arabidopsis* 28 kDa ferritin plays a main role in the regulation of cellular iron under oxidative stress in *Brasicca juncea* [55,56]. An oxidative burst is known to be one of the earliest events that take place in plants under stress [57].

Up to now, there have been very few studies on the resistance genes in rubber tree against powdery mildew infection, and the rubber tree’s defensive mechanisms to powdery mildew remain unclear. Qin *et al.* reported the cloning of an *Mlo* gene in a rubber tree in 2015 [3]. However, the *Mlo* gene expression did not change significantly during powdery mildew infection. Our DDRT-PCR analysis is the first to reveal the complex pattern of differential gene expression in the rubber tree during *O. heveae* powdery mildew infection. The identified *H. brasiliensis* genes involved in the infection process of *O. heveae* should provide a useful reference for developing new strategies to improve the resistance of rubber tree to powdery mildew disease.

## 4. Materials and Methods

### 4.1. Rubber Trees and O. heveae

The highly resistant rubber tree cultivar RRIC52 and the mildly susceptible cultivar Reyan 7-33-97 were both grown in plastic pots in the greenhouse at the experimental farm of Hainan University, Haikou, China. They were two-year-old grafted plants kept in the greenhouse until their leaves reached the suitable developmental stage for inoculation.

The powdery mildew pathogen *O. heveae* isolate HO-73 was isolated from the experimental farm and maintained on small Reyan 7-33-97 grafted plants.

### 4.2. Inoculation of Rubber Trees with O. heveae HO-73

The infected leaves of Reyan 7-33-97 grafted plants were shaken 24 h before inoculation to allow the formation of fresh conidial spores. For inoculation, conidia were dusted from infector rubber tree leaves to the leaves of the two cultivars. Plants were incubated in a growth chamber at 21–22 °C with 60%–70% humidity and a 16 h photoperiod for 120 h. Leaves from plants at different infection times (0, 12, 24, 72 and 120 h) were collected to analyze gene expression changes in response to powdery mildew infection. Leaf samples were also collected from uninoculated plants as controls. All samples were immediately frozen in liquid nitrogen and stored at −80 °C for total RNA extraction. Three biological replicates were used for all treatments.

Alcoholic trypan blue assay [58] was performed to analyze the *O. heveae* infection on tested plant leaves from RRIC52 and Reyan 7-33-97. The infected leaves were collected 120 h after inoculation. They were cleared for 10–60 min at 70 °C temperature in an ethanol-chloroform (75:25, *v*/*v*) mixture containing 0.15% trichloroacetic acid [59]. The leaf sections were stained with lactophenol–trypan blue (10 mL of lactic acid, 10 mL of glycerol, 10 g of phenol and 10 mg of trypan blue, dissolved in 10 mL of distilled water) for 5 min, and then examined under an Olympus BX-51 light microscope.

### 4.3. RNA Extraction and cDNA Synthesis

Total RNA was isolated from each leaf sample as described [60]. Genomic DNA was removed by DNase I digestion (RNase-free) (Thermo Scientific, Waltham, MA, USA). The RNA integrity was assessed by agarose gel electrophoresis. The concentration and quality of RNA were determined by the A260 absorbance and the A260/A280 ratio using a NanoDrop 2000 spectrophotometer (Thermo Scientific, Waltham, MA, USA). All RNA samples were adjusted to the same concentration in the subsequent reverse transcription reaction. Total RNA from powdery mildew-infected RRIC52 was used for DDRT-PCR. Total RNAs from powdery mildew-infected and control RRIC52 and Reyan7-33-97 plants were used for qRT-PCR.

### 4.4. Differential Display Analysis

mRNA differential display (DD) was performed essentially as previously described [61] with the total RNA of RRIC52. First-strand cDNA was synthesized using 2 μg total RNA as template by the RevertAid™ First Strand cDNA Synthesis Kit (Fermentas, Waltham, MA, USA) according to the manufacturer’s instruction. Three sets of anchored 3’-oligo (dT) primers (H-T11M, M = A/G/C) were used to synthesize first-strand cDNA. PCR amplification was performed using in combination with one reverse transcription step anchored primer and one arbitrary primer (B0301-B0326, Ap27-Ap34 in Supplementary Material, Table S4). The oligo (dT) primers and arbitrary primers were obtained from Sangon Biotech Corp (Shanghai, China). Each reaction mixture, in a total volume of 20 μL, contained 2 μL first strand cDNA, 2 mM each of dNTPs, 0.2 μM of random primers, 0.2 μM of anchored primer, and 1 unit of Taq DNA polymerase (Fermentas). The cycling parameters were as follows: 94 °C for 30 s, 40 °C for 2 min, 72 °C for 30 s for 40 cycles followed by 72 °C for 5 min. The amplified cDNAs were then separated on a 6% denaturing polyacrylamide gel.

### 4.5. Isolation of Differentially Displayed cDNA Bands from Agarose Gel

Compared with the control sample at 0 h, differentially expressed DNA bands were marked in the gel. They were excised from the gel and placed into 1.5-mL tubes with 100 μL dH_2_O (distilled water). The tubes were placed in a boiling water bath for 10 min. Each eluted DNA was used as the template for PCR re-amplification using the appropriate primer pairs. Successfully re-amplified cDNA fragments were electrophoresed on agarose gel and purified with a gel extraction kit (OMEGA, Norcross, GA, USA) and stored at −20 °C for reverse Northern analysis and subcloning.

### 4.6. Reverse Northern Dot Blot, Cloning, and Sequence Analysis

For the reverse Northern dot-blot analysis, the re-amplified and purified cDNAs (300 ng) were denatured with 0.1 M NaOH and neutralized with 0.1 M Tris–HCl. Each 1-μL sample was manually applied to one of five nylon membranes and baked for 30 min at 80 °C [62,63]. The cDNA pools of 0, 12, 24, 72 and 120 h were respectively labeled with DIG (DIG High Prime DNA Labeling and Detection Starter Kit II, Roche, Basel, Switzerlan) as probes to hybridize with the membrane blotted with the purified DNA bands according to the manufacturer’s instruction. An equal amount of each DIG-labeled cDNA mixture was denatured and separately hybridized to one of the five membranes according to the manufacturer’s instructions. The expression level of each cDNA for a differentially expressed gene was obtained from the ratio of the inoculated sample *versus* the control after normalizing to the expression of 18S rRNA.

After validation by DDRT-PCR, second round-PCR, and Reverse Northern dot blot analysis, the positive DNA bands were then cloned into a pMD19-T vector (Takara) and transformed into competent DH5α cells (TransGen Biotech, Beijing, China). Positive recombinant clones identified by PCR amplification were sequenced by BGI Biotech Co., Ltd. (Beijing, China). DNA sequences were analyzed against the National Center for Biotechnology Information (NCBI, Bethesda, MD, USA) database using the BLAST tool. Rahman *et al.* [64] reported the whole genome of *H. brasiliensis* clone RRIM 600 in the year 2013; we also have blasted (BLAST 2.2.29+) to analysis our 78 unique DD-derived ESTs in the presence genome.

### 4.7. Expression Validation by Real-Time Quantitative RT-PCR

qRT-PCR analyses were carried out to validate the putative differentially expressed genes that were likely involved in rubber tree defense mechanism against powdery mildew. PCR primers (Supplementary Material, Table S5) were designed using Primer 5.0 (Premier Biosoft International, Palo Alto, CA, USA). The gene expressions of two rubber tree cultivars that respond to powdery mildew differently were compared. We also have followed the expression of the candidate genes in both cultivars under control condition (no infection) during the whole time course.

The first-strand cDNA was synthesized from 2 μg total RNA in a total volume of 20 μL using the PrimeScript™ RT Reagent Kit (Takara). The PCR reaction was performed in a 10 μL reaction mixture containing 1× SYBR Premix EΧ Taq (Takara), 0.2 μM of each primer, and 15 ng cDNA per sample. The PCR reactions were performed with the following program: 95 °C for 30, 95 °C for 5 s, 60 °C for 20 s. and 72 °C for 20 s, for a total of 40 cycles in 384-well optical reaction plates with the CFX384 system (Bio-Rad Laboratories, Hercules, CA, USA). To validate all primers and amplifications, melting curves were examined for all qPCR products, and the amplification efficiency (*E*) of each primer set and correlation coefficient (*R*^2^) were calculated using the standard curve method. Data analysis was calculated by the Pfaffl method [10]. The housekeeping genes *RH8* and *YLS8*, which are stably expressed in the rubber tree [65], were both used as reference genes.

At least three independent biological replicates were performed for all the datasets. Each biological replicate was carried out in at least three technical replicates and the values were presented as mean ± S.E. from the results of three biological replicates. Student’s *t*-test (* indicates *p* < 0.05. **, *p* < 0.01) was used to determine the significant difference between the treatments.

## 5. Conclusions

In this study, we showed for the first time the identification of the responsive genes during powdery mildew infection in the rubber tree. We demonstrated the similar or different gene expression changes of eight such responsive genes between two rubber tree cultivars with different resistance levels by qRT-PCR. These eight genes include three fungal cell wall hydrolysis enzymes, a ferritin protein, a single-pass membrane protein, two regulation immune effectors, and a germin-like protein. Our study has provided the much-needed information to understand the powdery mildew defense mechanisms in the rubber tree.

## Supplementary Materials

Supplementary materials can be found at http://www.mdpi.com/1422-0067/17/2/181/s1.

## References

[1] Lertpanyasampatha M., Viboonjun U., Kongsawadworakul P., Chrestin H., Narangajavana J. (2014). Differential expression of microRNAs and their targets reveals a possible dual role in physiological bark disorder in rubber tree. J. Plant Physiol..

[2] Li D., Deng Z., Chen C., Xia Z., Wu M., He P., Chen S. (2010). Identification and characterization of genes associated with tapping panel dryness from hevea brasiliensis latex using suppression subtractive hybridization. BMC Plant Biol..

[3] Qin B., Zheng F., Zhang Y. (2015). Molecular cloning and characterization of a *Mlo* gene in rubber tree (*Hevea brasiliensis*). J. Plant Physiol..

[4] Limkaisang S., Kom-un S., Furtado E.L., Liew K.W., Salleh B., Sato Y., Takamatsu S. (2005). Molecular phylogenetic and morphological analyses of oidium heveae, a powdery mildew of rubber tree. Mycoscience.

[5] Manickavelu A., Kawaura K., Oishi K., Shin T., Kohara Y., Yahiaoui N., Keller B., Suzuki A., Yano K., Ogihara Y. (2010). Comparative gene expression analysis of susceptible and resistant near-isogenic lines in common wheat infected by *Puccinia triticina*. DNA Res..

[6] Liang P., Pardee A.B. (1992). Differential display of eukaryotic messenger RNA by means of the polymerase chain reaction. Science.

[7] Liang P. (2002). A decade of differential display. Biotechniques.

[8] Ruiyi Y.Z.W.S.F. (1992). Resistant behaviour of popular rubber clones in China to oidium heveae. Chin. J. Trop. Crops.

[9] EST database of GenBank. http://www.ncbi.nlm.nih.gov/biosample/?term=Library%20of%20differentially%20expressed%20%20%20%20%20%20%20%20%20%20%20%20%20%20%20%20%20transcripts%20in%20Hevea%20brasiliensis%20clone%20RRIC52%20inoculated%20%20%20%20%20%20%20%20%20%20%20%20%20%20%20%20%20with%20powdery%20mildew.

[10] Pfaffl M.W. (2001). A new mathematical model for relative quantification in real-time RT–PCR. Nucleic Acids Res..

[11] Elliott C., Zhou F., Spielmeyer W., Panstruga R., Schulze-Lefert P. (2002). Functional conservation of wheat and rice *Mlo* orthologs in defense modulation to the powdery mildew fungus. Mol. Plant-Microbe Interact..

[12] Barker C.L., Donald T., Pauquet J., Ratnaparkhe M., Bouquet A., Adam-Blondon A.-F., Thomas M., Dry I. (2005). Genetic and physical mapping of the grapevine powdery mildew resistance gene, *Run1*, using a bacterial artificial chromosome library. Theor. Appl. Genet..

[13] Donald T., Pellerone F., Adam-Blondon A.-F., Bouquet A., Thomas M., Dry I. (2002). Identification of resistance gene analogs linked to a powdery mildew resistance locus in grapevine. Theor. Appl. Genet..

[14] Coleman C., Copetti D., Cipriani G., Hoffmann S., Kozma P., Kovács L., Morgante M., Testolin R., di Gaspero G. (2009). The powdery mildew resistance gene *REN1* co-segregates with an NBS-LRR gene cluster in two central Asian grapevines. BMC Genet..

[15] Hoffmann S., di Gaspero G., Kovács L., Howard S., Kiss E., Galbács Z., Testolin R., Kozma P. (2008). Resistance to *Erysiphe necator* in the grapevine “Kishmish vatkana” is controlled by a single locus through restriction of hyphal growth. Theor. Appl. Genet..

[16] Xiao S., Ellwood S., Calis O., Patrick E., Li T., Coleman M., Turner J.G. (2001). Broad-spectrum mildew resistance in arabidopsis thaliana mediated by *RPW8*. Science.

[17] Dunwell J.M., Khuri S., Gane P.J. (2000). Microbial relatives of the seed storage proteins of higher plants: Conservation of structure and diversification of function during evolution of the cupin superfamily. Microbiol. Mol. Biol. Rev..

[18] Christensen A.B., Thordal-Christensen H., Zimmermann G., Gjetting T., Lyngkjær M.F., Dudler R., Schweizer P. (2004). The germinlike protein GLP4 exhibits superoxide dismutase activity and is an important component of quantitative resistance in wheat and barley. Mol. Plant-Microbe Interact..

[19] Wei Y., Zhang Z., Andersen C.H., Schmelzer E., Gregersen P.L., Collinge D.B., Smedegaard-Petersen V., Thordal-Christensen H. (1998). An epidermis/papilla-specific oxalate oxidase-like protein in the defence response of barley attacked by the powdery mildew fungus. Plant Mol. Biol..

[20] Gucciardo S., Wisniewski J.-P., Brewin N.J., Bornemann S. (2007). A germin-like protein with superoxide dismutase activity in pea nodules with high protein sequence identity to a putative rhicadhesin receptor. J. Exp. Bot..

[21] Schweizer P., Christoffel A., Dudler R. (1999). Transient expression of members of the germin-like gene family in epidermal cells of wheat confers disease resistance. Plant J..

[22] Zimmermann G., Bäumlein H., Mock H.-P., Himmelbach A., Schweizer P. (2006). The multigene family encoding germin-like proteins of barley. Regulation and function in basal host resistance. Plant Physiol..

[23] Godfrey D., Able A.J., Dry I.B. (2007). Induction of a grapevine germin-like protein (*VvGLP3*) gene is closely linked to the site of *Erysiphe necator* infection: A possible role in defense?. Mol. Plant-Microbe Interact..

[24] Van Loon L. (1985). Pathogenesis-related proteins. Plant Mol. Biol..

[25] Wolski E.A., Maldonado S., Daleo G.R., Andreu A.B. (2006). A novel α-1,3-glucan elicits plant defense responses in potato and induces protection against rhizoctonia solani AG-3 and *Fusarium solani* f. Sp. *eumartii*. Physiol. Mol. Plant Pathol..

[26] Shibuya N., Minami E. (2001). Oligosaccharide signalling for defence responses in plant. Physiol. Mol. Plant Pathol..

[27] Giannakis C., Bucheli C., Skene K., Robinson S., Scott N.S. (1998). Chitinase and β-1,3-glucanase in grapevine leaves: A possible defence against powdery mildew infection. Aust. J. Grape Wine Res..

[28] Libault M., Wan J., Czechowski T., Udvardi M., Stacey G. (2007). Identification of 118 arabidopsis transcription factor and 30 ubiquitin-ligase genes responding to chitin, a plant-defense elicitor. Mol. Plant-Microbe Interact..

[29] Rushton P.J., Somssich I.E., Ringler P., Shen Q.J. (2010). WRKY transcription factors. Trends Plant Sci..

[30] Pandey S.P., Roccaro M., Schön M., Logemann E., Somssich I.E. (2010). Transcriptional reprogramming regulated by WRKY18 and WRKY40 facilitates powdery mildew infection of arabidopsis. Plant J..

[31] Xu X., Chen C., Fan B., Chen Z. (2006). Physical and functional interactions between pathogen-induced arabidopsis WRKY18, WRKY40, and WRKY60 transcription factors. Plant Cell.

[32] Shen Q.-H., Saijo Y., Mauch S., Biskup C., Bieri S., Keller B., Seki H., Ülker B., Somssich I.E., Schulze-Lefert P. (2007). Nuclear activity of MLA immune receptors links isolate-specific and basal disease-resistance responses. Science.

[33] Šamaj J., Baluška F., Voigt B., Schlicht M., Volkmann D., Menzel D. (2004). Endocytosis, actin cytoskeleton, and signaling. Plant Physiol..

[34] Chen X., Irani N.G., Friml J. (2011). Clathrin-mediated endocytosis: The gateway into plant cells. Curr. Opin. Plant Biol..

[35] Thoma S., Hecht U., Kippers A., Botella J., de Vries S., Somerville C. (1994). Tissue-specific expression of a gene encoding a cell wall-localized lipid transfer protein from arabidopsis. Plant Physiol..

[36] Van Loon L., van Strien E. (1999). The families of pathogenesis-related proteins, their activities, and comparative analysis of PR-1 type proteins. Physiol. Mol. Plant Pathol..

[37] Roy-Barman S., Sautter C., Chattoo B.B. (2006). Expression of the lipid transfer protein Ace-AMP1 in transgenic wheat enhances antifungal activity and defense responses. Transgenic Res..

[38] Li X., Gasic K., Cammue B., Broekaert W., Korban S.S. (2003). Transgenic rose lines harboring an antimicrobial protein gene, Ace-AMP1, demonstrate enhanced resistance to powdery mildew (*Sphaerotheca pannosa*). Planta.

[39] Cao A., Xing L., Wang X., Yang X., Wang W., Sun Y., Qian C., Ni J., Chen Y., Liu D. (2011). Serine/threonine kinase gene *Stpk-V*, a key member of powdery mildew resistance gene *Pm21*, confers powdery mildew resistance in wheat. Proc. Natl. Acad. Sci. USA.

[40] Pedley K.F., Martin G.B. (2003). Molecular basis of Pto-mediated resistance to bacterial speck disease in tomato. Annu. Rev. Phytopathol..

[41] Yang Y., Shah J., Klessig D.F. (1997). Signal perception and transduction in plant defense responses. Genes Dev..

[42] Ryu H.-S., Han M., Lee S.-K., Cho J.-I., Ryoo N., Heu S., Lee Y.-H., Bhoo S.H., Wang G.-L., Hahn T.-R. (2006). A comprehensive expression analysis of the WRKY gene superfamily in rice plants during defense response. Plant Cell Rep..

[43] Trognitz F., Manosalva P., Gysin R., Niño-Liu D., Simon R., del Rosario Herrera M., Trognitz B., Ghislain M., Nelson R. (2002). Plant defense genes associated with quantitative resistance to potato late blight in *Solanum phureja* × Dihaploid *S. tuberosum* hybrids. Mol. Plant-Microbe Interact..

[44] Bézier A., Lambert B., Baillieul F. (2002). Study of defense-related gene expression in grapevine leaves and berries infected with *Botrytis cinerea*. Eur. J. Plant Pathol..

[45] Dao T., Linthorst H., Verpoorte R. (2011). Chalcone synthase and its functions in plant resistance. Phytochem. Rev..

[46] Mohanta T.K., Occhipinti A., Zebelo S.A., Foti M., Fliegmann J., Bossi S., Maffei M.E., Bertea C.M. (2012). Ginkgo biloba responds to herbivory by activating early signaling and direct defenses. PLoS ONE.

[47] Pezet R., Gindro K., Viret O., Spring J.-L. (2004). Glycosylation and oxidative dimerization of resveratrol are respectively associated to sensitivity and resistance of grapevine cultivars to downy mildew. Physiol. Mol. Plant Pathol..

[48] Treutter D. (2006). Significance of flavonoids in plant resistance: A review. Environ. Chem. Lett..

[49] Persans M.W., Wang J., Schuler M.A. (2001). Characterization of maize cytochrome p450 monooxygenases induced in response to safeners and bacterial pathogens. Plant Physiol..

[50] Qi X., Bakht S., Qin B., Leggett M., Hemmings A., Mellon F., Eagles J., Werck-Reichhart D., Schaller H., Lesot A. (2006). A different function for a member of an ancient and highly conserved cytochrome p450 family: From essential sterols to plant defense. Proc. Natl. Acad. Sci. USA.

[51] Whitbred J.M., Schuler M.A. (2000). Molecular characterization of cyp73a9 andcyp82a1 p450 genes involved in plant defense in pea. Plant Physiol..

[52] Kim Y.-C., Kim S.-Y., Paek K.-H., Choi D., Park J.M. (2006). Suppression of cacyp1, a novel cytochrome p450 gene, compromises the basal pathogen defense response of pepper plants. Biochem. Biophys. Res. Commun..

[53] Narusaka M., Seki M., Umezawa T., Ishida J., Nakajima M., Enju A., Shinozaki K. (2004). Crosstalk in the responses to abiotic and biotic stresses in arabidopsis: Analysis of gene expression in cytochrome p450 gene superfamily by cDNA microarray. Plant Mol. Biol..

[54] Deák M., Horváth G.V., Davletova S., Török K., Sass L., Vass I., Barna B., Király Z., Dudits D. (1999). Plants ectopically expressing the ironbinding protein, ferritin, are tolerant to oxidative damage and pathogens. Nat. Biotechnol..

[55] Arnaud N., Murgia I., Boucherez J., Briat J.-F., Cellier F., Gaymard F. (2006). An iron-induced nitric oxide burst precedes ubiquitin-dependent protein degradation for arabidopsis atfer1 ferritin gene expression. J. Biol. Chem..

[56] Yang J., Liu S., Yang X., Zhang M. (2012). Chloroplast-located BjFer1 together with anti-oxidative genes alleviate hydrogen peroxide and hydroxyl radical injury in cytoplasmic male-sterile *Brassica juncea*. Mol. Biol. Rep..

[57] Liu G., Greenshields D.L., Sammynaiken R., Hirji R.N., Selvaraj G., Wei Y. (2007). Targeted alterations in iron homeostasis underlie plant defense responses. J. Cell Sci..

[58] Keogh R., Deverall B., McLeod S. (1980). Comparison of histological and physiological responses to *Phakopsora pachyrhizi* in resistant and susceptible soybean. Trans. Br. Mycol. Soc..

[59] Wolf G., Fric F. (1981). A rapid staining method for *Erysiphe graminis* f. Sp. *hordei* in and on whole barley leaves with a protein-specific dye. Phytopathology.

[60] Xu J., Aileni M., Abbagani S., Zhang P. (2010). A reliable and efficient method for total RNA isolation from various members of spurge family (Euphorbiaceae). Phytochem. Anal..

[61] Liang P., Zhu W., Zhang X., Guo Z., O'Connell R.P., Averboukh L., Wang F., Pardee A.B. (1994). Differential display using one-base anchored oligo-dT primers. Nucleic Acids Res..

[62] Minglin L., Yuxiu Z., Tuanyao C. (2005). Identification of genes up-regulated in response to Cd exposure in *Brassica juncea* L. Gene.

[63] Xu J., Yin H., Wang W., Mi Q., Liao X., Li X. (2009). Identification of Cd-responsive genes of *Solanum nigrum* seedlings through differential display. Plant Mol. Biol. Rep..

[64] Rahman A.Y.A., Usharraj A.O., Misra B.B., Thottathil G.P., Jayasekaran K., Feng Y., Hou S., Ong S.Y., Ng F.L., Lee L.S. (2013). Draft genome sequence of the rubber tree *Hevea brasiliensis*. BMC Genom..

[65] Li H., Qin Y., Xiao X., Tang C. (2011). Screening of valid reference genes for real-time RT-PCR data normalization in *Hevea brasiliensis* and expression validation of a sucrose transporter gene *HbSUT3*. Plant Sci..

