# Supplementary material for: Identification of Powdery Mildew Responsive Genes in Hevea brasiliensis through mRNA Differential Display
Source: Int J Mol Sci. 2016 Jan 29;17(2):181. doi: 10.3390/ijms17020181 (PMC4783915; doi:10.3390/ijms17020181)
Supplement: Supplementary file 1 [file ijms-17-00181-s001.pdf]

Supplementary Materials: Identification of Powdery Mildew Responsive Genes in *Hevea brasiliensis* through mRNA Differential Display

Xiang Li, Zhenghong Bi, Rong Di, Peng Liang, Qiguang He, Wenbo Liu, Weiguo Miao and Fucong Zheng

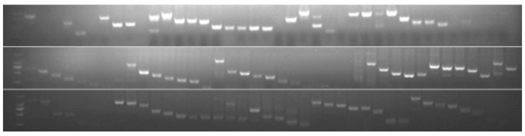

Figure S1. Electrophoresis of re-amplication PCR products of the differential expressed cDNA fragments.

Table S1. The fragment lengths, excised time point and the primer combination of 78 unique DD-derived ESTs.

ESTs	Primer Pair Used in DDRT-PCR	Size (bp)	0 h	12 h	24 h	72 h	120 h
HBOH1	T11C/B0322	1125			√	√	√
HBOH2	T11G/AP31	429	√	√	√		
HBOH3	T11A/B0308	759		√	√	√	√
HBOH4	T11G/B0314	685			√	√	
HBOH5	T11A/B0314	895				√	√
HBOH6	T11C/B0307	383					√
HBOH7	T11C/B0304	494	√				
HBOH8	T11C/B0304	203	√	√	√		
HBOH9	T11G/B0313	362					√
HBOH10	T11C/B0316	422					√
HBOH11	T11G/B0309	474	√				
HBOH12	T11C/B0317	492	√	√	√		
HBOH13	T11C/B0322	525				√	
HBOH14	T11G/B0308	251				√	
HBOH15	T11A/AP34	305				√	
HBOH17	T11C/B0308	541		√	√	√	
HBOH18	T11C/AP32	528	√	√	√		
HBOH19	T11C/B0317	527		√	√	√	√
HBOH21	T11C/B0322	1009		√			
HBOH22	T11C/B0322	847		√	√		
HBOH23	T11C/B0322	882		√	√		
HBOH24	T11C/B0323	804	√				
HBOH25	T11C/B0318	533		√			
HBOH26	T11A/B0324	712		√			
HBOH28	T11A/AP34	416		√	√	√	
HBOH29	T11G/B0308	399		√			
HBOH30	T11G/B0315	595		√			
HBOH31	T11G/AP32	217			√		
HBOH32	T11G/B0325	390		√	√	√	√
HBOH33	T11G/B0325	928		√			
HBOH35	T11G/B0325	703		√	√		
HBOH36	T11C/B0325	787					√
HBOH37	T11C/B0309	908		√	√		
HBOH38	T11C/B0310	876		√	√	√	
HBOH39	T11C/B0316	799		√			
HBOH40	T11C/AP34	665					√
HBOH41	T11C/B0317	481					√
HBOH42	T11C/B0323	357					√

Table S1. *Cont.*

ESTs	Primer Pair Used in DDRT-PCR	Size (bp)	0 h	12 h	24 h	72 h	120 h
HBOH43	T11C/B0324	919		√			
HBOH44	T11C/B0326	795		√	√		
HBOH45	T11C/B0318	701	√				
HBOH46	T11C/B0301	531	√				
HBOH47	T11C/B0315	415		√	√	√	
HBOH48	T11G/B0320	541	√				
HBOH49	T11G/B0304	471	√				
HBOH50	T11A/AP34	509		√	√		
HBOH51	T11G/B0325	501		√	√		
HBOH52	T11G/B0320	725	√				
HBOH53	T11C/B0318	623			√	√	
HBOH54	T11G/AP31	479		√			
HBOH55	T11C/AP33	885	√				
HBOH56	T11G/AP27	289		√	√	√	√
HBOH57	T11C/B0309	241		√	√	√	
HBOH60	T11G/B0307	454				√	
HBOH61	T11C/B0304	801		√	√		
HBOH62	T11C/B0317	354		√	√		
HBOH63	T11G/B0302	1019		√	√	√	√
HBOH64	T11G/B0304	338	√	√			
HBOH65	T11G/AP31	823				√	
HBOH66	T11G/AP28	492		√	√	√	√
HBOH67	T11G/B0303	636				√	√
HBOH68	T11A/B0325	460		√	√	√	√
HBOH69	T11A/B0320	492	√				
HBOH70	T11C/B0322	734		√	√	√	
HBOH72	T11A/B0318	707		√	√	√	√
HBOH73	T11G/B0310	284				√	
HBOH74	T11G/AP32	506		√	√	√	√
HBOH75	T11C/B0320	451		√	√	√	√
HBOH76	T11G/AP31	252				√	
HBOH77	T11G/B0319	519				√	
HBOH78	T11G/B0311	361				√	
HBOH79	T11G/AP27	766		√	√		
HBOH80	T11A/B0309	312				√	
HBOH81	T11C/AP29	282			√	√	√
HBOH82	T11C/B0319	721			√		
HBOH83	T11C/B0315	575		√	√	√	√
HBOH84	T11C/B0314	229		√	√	√	√
HBOH85	T11C/B0314	493		√	√	√	√

√, represents the time points what the ESTs were excised from.

Table S2. The 78 unique DD-derived ESTs mapping to the genome of *H. brasiliensis* clone RRIM 600.

Query	Target (Database Sequence or Cluster Centroid)	Percent Identity	Alignment Length	Number of Mismatches	Number of Gap Opens	1-Based Position of Start in Query	1-Based Position of End in Query	1-Based Position of Start in Target	1-Based Position of End in Target	*E*-value Calculated Using Karlin–Altschul Statistics	Bit Score Calculated Using Karlin–Altschul Statistics
HBOH1	gi|461680035|gb|KB613559.1|	97.7	261	4	2	7	266	11,354	11,095	8×10^{-124}	448
HBOH1	gi|461680035|gb|KB613559.1|	91.08	269	11	13	830	1085	9527	9259	6×10^{-95}	351
HBOH1	gi|461680035|gb|KB613559.1|	94.57	184	10	0	651	834	9783	9600	7×10^{-75}	285
HBOH1	gi|461680035|gb|KB613559.1|	98.01	151	3	0	501	651	10,350	10,200	3×10^{-68}	263
HBOH1	gi|461680035|gb|KB613559.1|	95.83	96	3	1	259	354	10,997	10,903	2×10^{-35}	154
HBOH1	gi|461680035|gb|KB613559.1|	100	79	0	0	422	500	10,553	10,475	3×10^{-33}	147
HBOH1	gi|461680035|gb|KB613559.1|	98.59	71	1	0	353	423	10,820	10,750	4×10^{-27}	126
HBOH2	gi|461667107|gb|KB617213.1|	99.29	425	3	0	5	429	17,702	18,126	0	769
HBOH3	gi|461663660|gb|KB618165.1|	100	426	0	0	1	426	671	1096	0	787
HBOH3	gi|461663660|gb|KB618165.1|	99.47	190	0	1	571	759	1449	1638	7×10^{-93}	344
HBOH3	gi|461663660|gb|KB618165.1|	100	150	0	0	424	573	1182	1331	7×10^{-73}	278
HBOH4	gi|461658666|gb|KB619572.1|	99.55	223	1	0	463	685	19,096	19,318	8×10^{-112}	407
HBOH4	gi|461658666|gb|KB619572.1|	100	90	0	0	182	271	17,472	17,561	2×10^{-39}	167
HBOH4	gi|461658666|gb|KB619572.1|	100	84	0	0	39	122	17,134	17,217	3×10^{-36}	156
HBOH4	gi|461658666|gb|KB619572.1|	100	66	0	0	399	464	18,903	18,968	3×10^{-26}	122
HBOH4	gi|461658666|gb|KB619572.1|	100	65	0	0	271	335	18,442	18,506	1×10^{-25}	121
HBOH4	gi|461658666|gb|KB619572.1|	97.06	68	2	0	334	401	18,649	18,716	6×10^{-24}	115
HBOH4	gi|461658666|gb|KB619572.1|	96.92	65	2	0	119	183	17,291	17,355	3×10^{-22}	110
HBOH4	gi|461658666|gb|KB619572.1|	97.37	38	1	0	1	38	17,001	17,038	0.000000006	65.8
HBOH5	gi|461667335|gb|KB617140.1|	100	282	0	0	1	282	16,805	17,086	4×10^{-146}	521
HBOH5	gi|461667335|gb|KB617140.1|	96.82	157	5	0	277	433	17,159	17,315	2×10^{-68}	263
HBOH5	gi|461667335|gb|KB617140.1|	97	100	1	2	429	527	17,390	17,488	2×10^{-39}	167
HBOH6	gi|461626191|gb|KB628913.1|	100	378	0	0	4	381	59,060	59,437	0	699
HBOH7	gi|461632587|gb|KB626968.1|	98.87	265	3	0	4	268	66,147	66,411	6×10^{-132}	473
HBOH7	gi|461632587|gb|KB626968.1|	98.67	226	3	0	267	492	66,503	66,728	3×10^{-110}	401
HBOH10	gi|461650867|gb|KB621653.1|	99.53	422	2	0	1	422	40,634	41,055	0	769
HBOH11	gi|461635840|gb|KB626009.1|	97.51	482	2	1	1	472	21,385	20,904	0	815
HBOH12	gi|461665307|gb|KB617694.1|	87.88	396	35	7	28	420	27,721	27,336	7×10^{-126}	453
HBOH13	gi|461670953|gb|KB616103.1|	94.18	292	14	1	234	525	133,280	132,992	2×10^{-122}	442
HBOH13	gi|461670953|gb|KB616103.1|	93.19	235	16	0	1	235	133,749	133,515	1×10^{-93}	346
HBOH14	gi|461670478|gb|KB616225.1|	100	216	0	0	36	251	125,418	125,633	5×10^{-110}	399
HBOH14	gi|461670478|gb|KB616225.1|	100	39	0	0	1	39	125,300	125,338	1×10^{-11}	73.1
HBOH15	gi|461642630|gb|KB623900.1|	95.24	210	10	0	96	305	23,376	23,585	6×10^{-90}	333
HBOH15	gi|461642630|gb|KB623900.1|	94.76	210	11	0	96	305	9465	9674	3×10^{-88}	327
HBOH15	gi|461642630|gb|KB623900.1|	100	96	0	0	1	96	9197	9292	3×10^{-43}	178
HBOH15	gi|461642630|gb|KB623900.1|	94.32	88	5	0	9	96	23,116	23,203	2×10^{-30}	135
HBOH17	gi|461662976|gb|KB618358.1|	100	541	0	0	1	541	62,960	62,420	0	1000
HBOH18	gi|461676424|gb|KB614594.1|	99.43	523	3	0	1	523	47,747	48,269	0	950
HBOH19	gi|461650565|gb|KB621723.1|	99.43	524	3	0	1	524	184,387	183,864	0	952
HBOH20	gi|461668646|gb|KB616741.1|	99.56	228	1	0	1	228	57,887	58,114	4×10^{-115}	416
HBOH21	gi|461633928|gb|KB626561.1|	98.32	1,011	9	6	2	1,009	28,639	29,644	0	1766
HBOH22	gi|461642726|gb|KB623869.1|	100	337	0	0	511	847	86,250	86,586	9×10^{-177}	623

Table S1. *Cont.*

Query	Target (Database Sequence or Cluster Centroid)	Percent Identity	Alignment Length	Number of Mismatches	Number of Gap Opens	1-Based Position of Start in Query	1-Based Position of End in Query	1-Based Position of Start in Target	1-Based Position of End in Target	*E*-value Calculated Using Karlin–Altschul Statistics	Bit Score Calculated Using Karlin–Altschul Statistics
HBOH22	gi|461642726|gb|KB623869.1|	100	214	0	0	1	214	84,551	84,764	2×10^{-108}	396
HBOH22	gi|461642726|gb|KB623869.1|	92.31	143	7	2	210	350	84,843	84,983	2×10^{-49}	200
HBOH22	gi|461642726|gb|KB623869.1|	99.03	103	1	0	316	418	85,402	85,504	5×10^{-45}	185
HBOH22	gi|461642726|gb|KB623869.1|	100	100	0	0	414	513	85,953	86,052	5×10^{-45}	185
HBOH23	gi|461659473|gb|KB619364.1|	90.69	881	76	6	1	877	77,526	78,404	0	1168
HBOH24	gi|461661228|gb|KB618878.1|	99.32	735	3	1	1	735	44,768	44,036	0	1328
HBOH25	gi|461662300|gb|KB618559.1|	99.06	533	5	0	1	533	20,175	20,707	0	957
HBOH26	gi|461662900|gb|KB618379.1|	98.67	679	9	0	34	712	71,698	71,020	0	1205
HBOH26	gi|461662900|gb|KB618379.1|	100	36	0	0	1	36	71,843	71,808	0.000000002	67.6
HBOH27	gi|461652363|gb|KB621251.1|	94.21	242	3	2	7	247	76,677	76,908	8×10^{-98}	359
HBOH28	gi|461672116|gb|KB615771.1|	97.53	81	2	0	126	206	83,267	83,187	2×10^{-31}	139
HBOH28	gi|461672116|gb|KB615771.1|	96.39	83	3	0	43	125	83,444	83,362	7×10^{-31}	137
HBOH28	gi|461672116|gb|KB615771.1|	93.33	45	3	0	1	45	83,687	83,643	9×10^{-10}	67.6
HBOH29	gi|461669880|gb|KB616391.1|	100	143	0	0	1	143	214,855	214,713	3×10^{-69}	265
HBOH30	gi|461629675|gb|KB627871.1|	99.83	595	1	0	1	595	33,419	32,825	0	1094
HBOH30	gi|461629675|gb|KB627871.1|	94.85	136	5	2	58	192	29,999	29,865	6×10^{-53}	211
HBOH31	gi|461667138|gb|KB617199.1|	96.35	219	5	2	1	217	12,200	12,417	2×10^{-97}	357
HBOH32	gi|461665655|gb|KB617602.1|	99.49	390	2	0	1	390	38,417	38,806	0	710
HBOH33	gi|461670666|gb|KB616176.1|	98.65	739	10	0	1	739	978	240	0	1310
HBOH35	gi|461684913|gb|KB612472.1|	97.12	347	10	0	350	696	123,319	123,665	1×10^{-165}	586
HBOH36	gi|461642278|gb|KB624012.1|	87.59	274	28	6	446	717	35,038	34,769	2×10^{-83}	313
HBOH37	gi|461626111|gb|KB628940.1|	99.23	909	6	1	1	908	11,843	12,751	0	1639
HBOH38	gi|461630644|gb|KB627588.1|	88.79	330	36	1	309	638	9505	9833	1×10^{-110}	403
HBOH38	gi|461630644|gb|KB627588.1|	94.94	178	9	0	631	808	11,719	11,896	2×10^{-73}	279
HBOH39	gi|461630644|gb|KB627588.1|	88.79	330	36	1	240	569	9833	9505	1×10^{-110}	403
HBOH39	gi|461630644|gb|KB627588.1|	94.94	178	9	0	70	247	11,896	11,719	2×10^{-73}	279
HBOH40	gi|461666443|gb|KB617392.1|	99.24	661	5	0	5	665	27,338	26,678	0	1194
HBOH41	gi|461636441|gb|KB625824.1|	80.51	395	46	17	45	412	93,516	93,906	6×10^{-72}	274
HBOH42	gi|461662300|gb|KB618559.1|	100	228	0	0	1	228	20,707	20,480	1×10^{-116}	422
HBOH42	gi|461662300|gb|KB618559.1|	100	131	0	0	227	357	20,305	20,175	1×10^{-62}	243
HBOH43	gi|461662300|gb|KB618559.1|	99.64	548	2	0	372	919	20,160	20,707	0	1002
HBOH44	gi|461660551|gb|KB619071.1|	98.57	490	1	1	312	795	22,456	21,967	0	861
HBOH44	gi|461660551|gb|KB619071.1|	100	158	0	0	1	158	24,610	24,453	3×10^{-77}	292
HBOH44	gi|461660551|gb|KB619071.1|	99.35	154	1	0	157	310	24,003	23,850	2×10^{-73}	279
HBOH45	gi|461670680|gb|KB616173.1|	81.57	369	41	16	6	372	18,876	19,219	2×10^{-73}	279
HBOH45	gi|461670680|gb|KB616173.1|	80.21	283	37	10	422	699	19,219	19,487	7×10^{-48}	195
HBOH46	gi|461638564|gb|KB625154.1|	99.62	531	2	0	1	531	31,990	31,460	0	970
HBOH47	gi|461635845|gb|KB626008.1|	99.52	413	2	0	1	413	25,955	26,367	0	752
HBOH48	gi|461649598|gb|KB621971.1|	99.82	541	1	0	1	541	17,060	16,520	0	994
HBOH49	gi|461668827|gb|KB616693.1|	99.36	469	3	0	3	471	45,201	45,669	0	850
HBOH50	gi|461659752|gb|KB619288.1|	99.4	504	3	0	6	509	76,501	75,998	0	915
HBOH51	gi|461639141|gb|KB624975.1|	99.75	407	0	1	96	501	11,656	12,062	0	745

Table S1. *Cont.*

Query	Target (Database Sequence or Cluster Centroid)	Percent Identity	Alignment Length	Number of Mismatches	Number of Gap Opens	1-Based Position of Start in Query	1-Based Position of End in Query	1-Based Position of Start in Target	1-Based Position of End in Target	*E*-value Calculated Using Karlin–Altschul Statistics	Bit Score Calculated Using Karlin–Altschul Statistics
HBOH52	gi|461676234|gb|KB614643.1|	99.21	127	1	0	1	127	72,362	72,236	2×10^{-58}	230
HBOH53	gi|461664453|gb|KB617935.1|	99.42	519	3	0	105	623	16,529	17,047	0	942
HBOH54	gi|461669887|gb|KB616389.1|	94.95	475	17	2	8	479	14,984	15,454	0	737
HBOH55	gi|461684469|gb|KB612547.1|	98.18	603	10	1	1	603	21,931	22,532	0	1051
HBOH55	gi|461684469|gb|KB612547.1|	98.95	285	3	0	601	885	22,636	22,920	8×10^{-143}	510
HBOH56	gi|461644365|gb|KB623362.1|	99.65	289	1	0	1	289	115,640	115,928	7×10^{-149}	529
HBOH60	gi|461670953|gb|KB616103.1|	98.89	359	4	0	96	454	134,235	134,593	0	641
HBOH60	gi|461670953|gb|KB616103.1|	97.98	99	2	0	1	99	133,962	134,060	2×10^{-41}	172
HBOH61	gi|461657407|gb|KB619943.1|	97.65	298	7	0	1	298	72,794	73,091	2×10^{-143}	512
HBOH61	gi|461657407|gb|KB619943.1|	98.82	255	2	1	297	550	73,269	73,523	1×10^{-125}	453
HBOH62	gi|461624260|gb|KB629400.1|	100	304	0	0	51	354	10,367	10,064	8×10^{-159}	562
HBOH62	gi|461624260|gb|KB629400.1|	100	51	0	0	1	51	10,516	10,466	4×10^{-18}	95.3
HBOH63	gi|461679985|gb|KB613572.1|	99.9	1018	1	0	1	1018	5302	4285	0	1875
HBOH64	gi|461675532|gb|KB614828.1|	88.76	89	6	3	225	309	3081	3169	2×10^{-21}	106
HBOH65	gi|461684073|gb|KB612612.1|	97.69	736	16	1	1	735	139,701	140,436	0	1264
HBOH65	gi|461684073|gb|KB612612.1|	92.35	405	30	1	388	791	148,249	148,653	3×10^{-162}	575
HBOH66	gi|461673775|gb|KB615316.1|	99.61	256	1	0	1	256	2436	2181	3×10^{-130}	468
HBOH66	gi|461673775|gb|KB615316.1|	100	236	0	0	257	492	2082	1847	7×10^{-121}	436
HBOH67	gi|461642038|gb|KB624091.1|	98.12	638	10	1	1	636	38,885	39,522	0	1110
HBOH69	gi|461663770|gb|KB618131.1|	100	492	0	0	1	492	15,832	15,341	0	909
HBOH70	gi|461644400|gb|KB623349.1|	99.71	346	0	1	207	551	96,468	96,123	1×10^{-179}	632
HBOH70	gi|461644400|gb|KB623349.1|	100	212	0	0	1	212	97,539	97,328	2×10^{-107}	392
HBOH70	gi|461644400|gb|KB623349.1|	100	192	0	0	543	734	96,023	95,832	3×10^{-96}	355
HBOH72	gi|461680666|gb|KB613387.1|	100	707	0	0	1	707	1441	2147	0	1306
HBOH73	gi|461640719|gb|KB624502.1|	100	44	0	0	241	284	3901	3858	2×10^{-14}	82.4
HBOH74	gi|461654326|gb|KB620738.1|	99.41	506	3	0	1	506	12,390	11,885	0	918
HBOH75	gi|461677564|gb|KB614257.1|	100	451	0	0	1	451	3381	3831	0	833
HBOH76	gi|461686912|gb|KB612173.1|	98.92	186	2	0	30	215	12,271	12,456	5×10^{-90}	333
HBOH78	gi|461623133|gb|KB629648.1|	100	361	0	0	1	361	6500	6860	0	667
HBOH79	gi|461670656|gb|KB616178.1|	91.61	322	27	0	209	530	10,365	10,686	2×10^{-123}	446
HBOH79	gi|461670656|gb|KB616178.1|	90.69	204	19	0	5	208	9803	10,006	3×10^{-71}	272
HBOH79	gi|461670656|gb|KB616178.1|	97.01	134	4	0	530	663	10,797	10,930	3×10^{-57}	226
HBOH79	gi|461670656|gb|KB616178.1|	96.97	99	3	0	668	766	11,050	11,148	2×10^{-39}	167
HBOH80	gi|461659070|gb|KB619472.1|	100	312	0	0	1	312	163,011	162,700	3×10^{-163}	577
HBOH81	gi|461670430|gb|KB616239.1|	100	277	0	0	6	282	13,200	13,476	7×10^{-144}	512
HBOH82	gi|461642576|gb|KB623916.1|	99.86	721	1	0	1	721	49,489	50,209	0	1327
HBOH83	gi|461642545|gb|KB623925.1|	99.65	575	2	0	1	575	48,740	49,314	0	1051

Table S3. The redundancy analysis of 78 unique DD-derived ESTs.

Query	Target	Percent Identity	Alignment Length	Number of Mismatches	Number of Gap Opens	1-Based Position of Start in Query	1-Based Position of End in Query	1-Based Position of Start in Target	1-Based Position of End in Target	*E*-Value Calculated Using Karlin–Altschul Statistics	Bit Score Calculated Using Karlin–Altschul statistics
HBOH25	HBOH43	98.87	533	6	0	1	533	387	919	0	952
HBOH25	HBOH42	100	228	0	0	306	533	228	1	2.00×10^{-120}	422
HBOH25	HBOH42	99.24	131	1	0	1	131	357	227	8.00×10^{-65}	237
HBOH38	HBOH39	99.47	568	3	0	309	876	569	2	0	1033
HBOH38	HBOH39	99.15	235	2	0	1	235	799	565	9.00×10^{-121}	424
HBOH39	HBOH38	99.47	568	3	0	2	569	876	309	0	1033
HBOH39	HBOH38	99.15	235	2	0	565	799	235	1	8.00×10^{-121}	424
HBOH42	HBOH25	100	228	0	0	1	228	533	306	1.00×10^{-120}	422
HBOH42	HBOH25	99.24	131	1	0	227	357	131	1	5.00×10^{-65}	237
HBOH42	HBOH43	99.56	228	1	0	1	228	919	692	6.00×10^{-119}	416
HBOH42	HBOH43	100	131	0	0	227	357	517	387	1.00×10^{-66}	243
HBOH43	HBOH25	98.87	533	6	0	387	919	1	533	0	952
HBOH43	HBOH64	99.7	338	1	0	18	355	1	338	1.00×10^{-179}	619
HBOH43	HBOH42	99.56	228	1	0	692	919	228	1	2.00×10^{-118}	416
HBOH64	HBOH43	99.7	338	1	0	1	338	18	355	4.00×10^{-180}	619

Table S4. Sequences of the 5′ arbitrary primers and 3′ anchored primers that were used in DDRT-PCR.

Primer Sequences					
Arbitrary primer Sequences (5′ to 3′)					
B0301	TACAACGAGG	B0313	GTTTTCGCAG	B0325	GATCATAGCG
B0302	TGGATTGGTC	B0314	GATCAAGTCC	B0326	GATCTAAGGC
B0303	CTTTCTACCC	B0315	GATCCAGTAC	AP27	AAGCTTGATTGCC
B0304	TTTTGGCTCC	B0316	GATCACGTAC	AP28	AAGCTTCGACTGT
B0305	GGAACCAATC	B0317	GATCTGACAC	AP29	AAGCTTTGGTCAG
B0306	AAACTCCGTC	B0318	GATCTCAGAC	AP30	AAGCTTCTCAACG
B0307	TCGATACAGG	B0319	GATCATAGCC	AP31	AAGCTTAGTAGGC
B0308	TGGTAAAGGG	B0320	GATCAATCGC	AP32	AAGCTTGCACCAT
B0309	TCGGTCATAG	B0321	GATCTAACCG	AP33	AAGCTTAACGAGG
B0310	GGTACATTGG	B0322	GATCGCATTG	AP34	AAGCTTTTACCGC
B0311	TACCTAAGCG	B0323	GATCTGACTG		
B0312	CTGCTTGATG	B0324	GATCATGGTC		
Anchor primer Sequences (5′ to 3′)					
B0327	AAGCTTTTTTTTTTA				
B0328	AAGCTTTTTTTTTTC				
B0329	AAGCTTTTTTTTTTG				

Table S5. Gene-specific primers for analysis by real-time PCR.

Primers(QF/QR)	Sequences (5′ to 3′)	Primer Efficiency	Reference
RH8(reference)-QF	TCACAGGGTTGGTAGATCAG	1.99	[65]
RH8(reference)-QR	CCAAGCTCTTGCTCAATCC		
YLS8(reference)-QF	CCTCGTCGTCATCCGATTC	1.98	[65]
YLS8(reference)-QR	CAGGCACCTCAGTGATGTC		
HBOH3-QF	CCTCATCAGCAGAACAATGTGGAA	1.94	
HBOH3-QR	ACGGTGTGGGGGTGGA		
HBOH4-QF	GCAATAAACGAGCAGGTCAAT	2.04	
HBOH4-QR	CTCTCTTTCTTCTTCACTGGATTC		
HBOH5-QF	TGCAGCTTTCTCGCCTCTCA	1.91	
HBOH5-QR	ATTCAACTGGTCTGGCACTGTCTC		
HBOH7-QF	TCTTCCCTGCTGGAGTTGG	1.93	
HBOH7-QR	GGTAGAGAATGCGATTGCGT		
HBOH9-QF	GTCCCACCTCCATCTTCCCA	1.95	
HBOH9-QR	CAGCCCGTGTTTGCCTCA		
HBOH13-QF	CACTGAAACCTTCCTCCCCAACT	2.01	
HBOH13-QR	CGGCGGCTATGGACTATTCTTATTC		
HBOH14-QF	CGGAGGATAAATCATTACTGGTTGC	1.95	
HBOH14-QR	GTTTTTCCTTGTCATCTATGGGGCT		
HBOH15-QF	TTCTGGCTTTGTCTTCCTCCTTT	1.94	
HBOH15-QR	GCCCTGGTATTTTATCAACATTTA		

QF, represents the Forward prime for qRT-PCR; QR, represents the Reversed prime for qRT-PCR.
